# Extracorporeal Membrane Oxygenation (ECMO) Training Program in A Pediatric Cardiac Intensive Care Unit: An 8-Year Single-Center Experience in Argentina

**DOI:** 10.21470/1678-9741-2021-0560

**Published:** 2022

**Authors:** María Luisa Pilan, Mariela Krynski, Guillermo Eduardo Moreno, Gisela M. Ponce, Ricardo E. Rodríguez, Estela R. Domínguez, Carlos Javier Cornelis, Maria Mercedes Montonati, Ana Miriam Lenz, Maria Belen Desocio, Nélida N. Bravo, Juan Carlos Vassallo, Silvia N. Santos, Gabriela V. Rodríguez, Barbara Cordisco, Maria J. Althabe

**Affiliations:** 1 Pediatric Cardiac Intensive Care Unit, Hospital de Pediatría J. P. Garrahan, Buenos Aires, Argentina; 2 Cardiovascular Surgery, Hospital de Pediatría J. P. Garrahan, Buenos Aires, Argentina; 3 Simulation Center, Hospital de Pediatría J. P. Garrahan, Buenos Aires, Argentina

**Keywords:** Extracorporeal Membrane Oxygenation, Simulation, Continuing Education, Training Courses, Education, Intensive Care Units, Demographic Data

## Abstract

**Introduction:**

Extracorporeal membrane oxygenation (ECMO) for temporary cardiopulmonary support is one of the most intense and technologically complex therapies offered in medicine. It is a high-risk procedure that requires specific knowledge and technical skills to perform it with good results.

**Objective:**

The main goal of this study is to describe our extracorporeal membrane oxygenation (ECMO) training program based on the study of specialized nurses and physicians of a simulation teaching experience, conducted in a pediatric cardiac intensive care unit. The program was developed as a theoretical-practical course with final exam and annual maintenance training sessions, caring for ECMO patients, its implementation and results.

**Methods:**

A descriptive study for registered nurses, intensivists, and cardiac surgeons. A self-administered, anonymous, and voluntary survey was conducted to assess the long-term perception about the program. Demographic data to describe the population was required, and questions about satisfaction and confidence in acquired skills and competences were asked. A descriptive statistical analysis was performed; patient survival and complications were compared before and after ECMO program using chi-square test, and P<0.05 was considered statistically significant.

**Results:**

Twenty-four training courses were performed for 68 professionals. More than 88% of the professionals considered the course components to be adequate and complete; and 94% felt trained to manage the ECMO circuit. Most valued activities were workshops and clinical cases. Since the implementation of the training program, 88 patients were assisted, with a survival rate at discharge of 58%, higher than in the previous period (P=0.03).

**Conclusion:**

More than 80% of the professionals considered the workshops and simulations as the most useful components. Reliance on the circuit care was higher than in training problem scenarios. Since 2013 we assisted 88 patients on ECMO, with a survival rate at discharge of 58%, within international standards results.

**Table:** 

Abbreviations, Acronyms & Symbols	
ECMO	= Extracorporeal membrane oxygenation
ELSO	= Extracorporeal Life Support Organization
IRB	= Institutional Review Board
IQR	= Interquartile range
PCICU	= Pediatric cardiac intensive care unit

## INTRODUCTION

Extracorporeal membrane oxygenation (ECMO) for temporary cardiopulmonary support is one of the most intense and technologically complex therapies offered in medicine. Nowadays, it is considered a standard of treatment in congenital heart defects repair and 1-2% of the patients are supported with it during postoperative care^[1]^. It is a high-risk procedure that requires specific knowledge and technical skills to perform it with good results. At our hospital, 600 patients per year undergo congenital heart surgery with a mortality rate of >5%^[2]^. We developed our ECMO program in 2006 and, together with surgeons, perfusionists, nurses and cardiac intensivists, cared for 123 ECMO patients with a 54% survival rate at discharge^[3]^. In 2012, facing the lack of perfusionists, we were forced to develop a different mode of care, in which control and monitoring of ECMO circuit is performed by trained nurses and physicians supported by specialist nurses on-call who provide support on a 24/7 basis. Each patient is cared for 24 hours a day by two professionals, one of them for the circuit, pump control and monitoring, and the other for specific nursing care. To implement this mode of care, we developed protocols for ECMO patient care and a training program following the Extracorporeal Life Support Organization (ELSO) guidelines^[4]^. We considered that simulation-based training would be an appropriated teaching tool to acquire the necessary competences to bring qualified care for these patients.

The objective of this study is to describe the training program and its implementation, to analyze the teaching performance from the perspective of the participants, and to review clinical outcomes with the new mode of care after 8 years of its implementation.

## METHODS

**Design:** Descriptive study about a simulation teaching experience.

**Setting:** Pediatric cardiac intensive care unit.

### Intervention

The training program began in 2013 and was addressed to pediatric cardiac intensive care unit (PCICU) physicians and nurses. We developed a theoretical-practical course with a final exam, and periodical maintenance training sessions. In-person and online lessons, readings and videos were available in our virtual campus, followed by workshops to address ECMO circuit operation, and simulation of emergency scenarios performed in our Simulation Center. Participant requirements were to be a pediatric intensive care certificated physician, pediatric critical care registered nurse, or perfusionist with more than two years’ experience in our unit. Minor changes were made along the years without affecting the basic structure of the training model organized into basic course, advanced course, and periodic training sessions to maintain skills. The most relevant chapters of the ELSO training manual were translated for better understanding and used as mandatory bibliography.

**Fig. 1:**
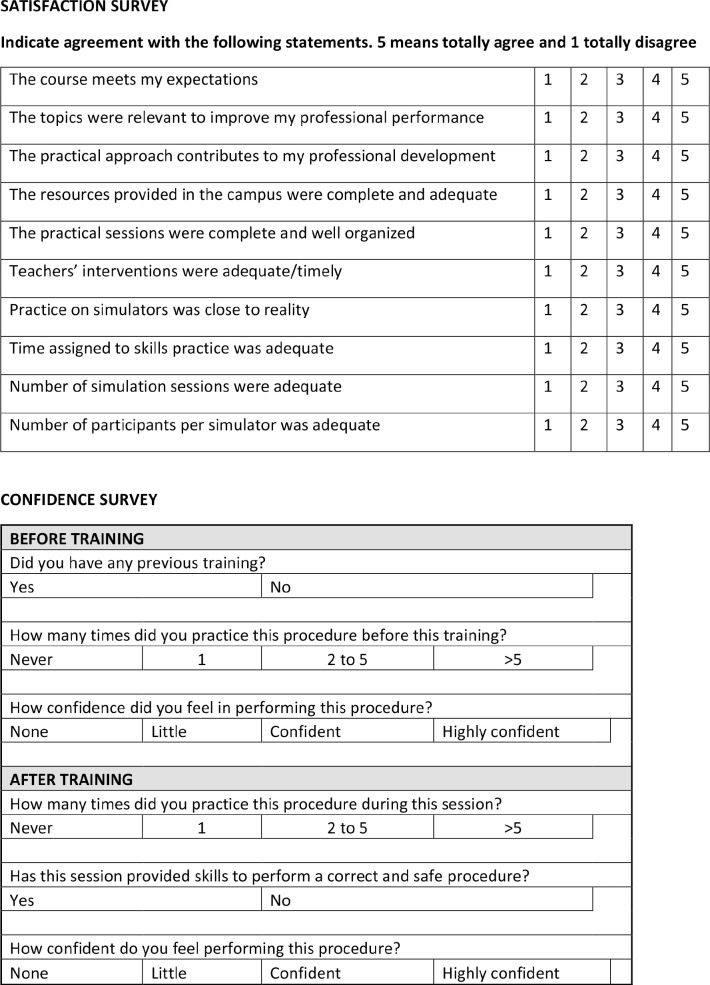
Survey after each teaching activity.

To complete the training course, professionals were required to pass a final theoretical multiple-choice exam and a practical exam of acquired skills in two simulation scenarios developed with the team. A detailed curriculum of the program is outlined in Appendix 1. For quality assessment, at the end of each course we conducted two satisfaction and self-confidence surveys (Figure 1).

**Fig. 2:**
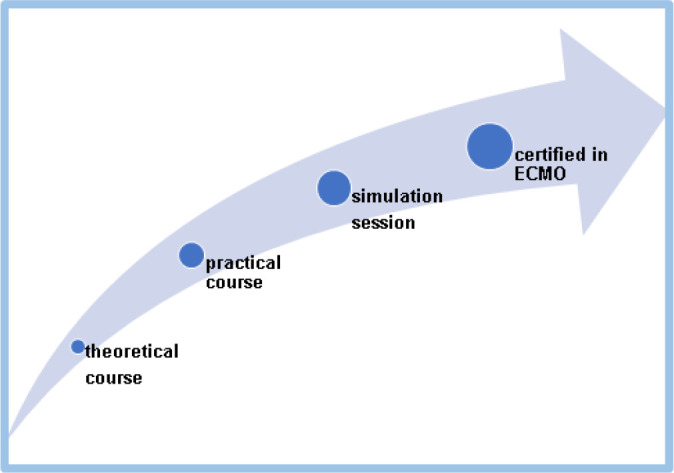
ECMO training program stages.

Once approved, the professionals of the course joined the ECMO team, with increasing responsibilities in patient connection and disconnection, circuit monitoring, replacement, and patient transport. Members of the ECMO team must repeat the advanced course annually and take care for at least two ECMO patients per year (credentialing maintenance) (Figure 2).

To evaluate the long-term perception of the training program, a self-administered, anonymous, and voluntary survey was conducted to all nurses and physicians of the PCICU (RedCap) after 8 years of continuing education activities. A small set of demographic data to describe the population was required, and questions addressed to evaluate satisfaction and confidence in acquired skills and competences throughout the program were asked. The educational resources evaluated in a 4-point Likert scale included: didactic material, theoretical classes, workshops, clinical cases, and simulation sessions. A reminder after two weeks to maximize the response rate was emailed. The survey is outlined in Appendix 2.

This study was approved by the Institutional Review Board (IRB) of our hospital, who waived the need for informed consent (protocol investigation No. 1272).

To assess the impact of the program in patient outcomes, we compared ECMO patient survival in two periods: 2006-2012 and 2013-2020. Descriptive and statistical analyses were performed; results are expressed as absolute values and percentages or median and interquartile range (IQR) as appropriated. Results were compared by chi-square tests, considering a *P*<0.05 as significant.

## RESULTS

Overall, we performed 24 courses (9 basic and 15 advanced), and different activities related to maintenance training. Seminars with specialists from other centers, technology updating workshops, and organization of the 1^st^ interactive symposium with simulation scenarios for specific issues, workshops on surgical management and specific nursing care for children on ECMO.

Sixty-eight professionals attended the courses: 32 intensivists and 36 critical care registered nurses, all of them with more than 2 years of experience in the PCICU. Sixty percent of them (41) are still participating in the ECMO maintenance training program.

Regarding the survey results, 68% of the responses (70/102) were received. Eighty-eight percent of the respondents attended to ECMO training activities from the beginning of the program, and currently 50% of them are members of the ECMO team.

**Fig. 3:**
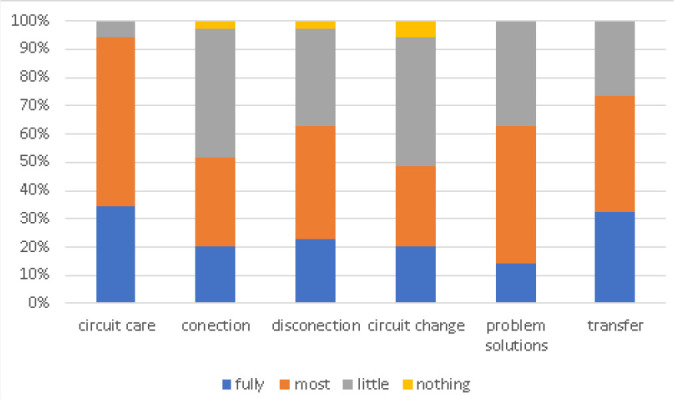
Perceived skills to solve situations after training. Confidence in ECMO circuit care was higher than in other situations.

**Fig. 4:**
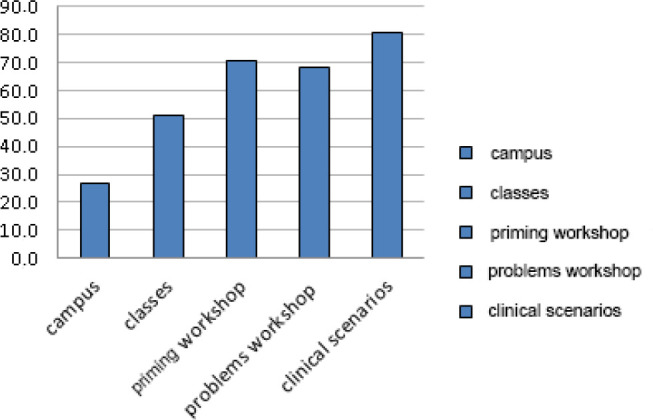
Course components usefulness assessment (%). The most valued components were clinical cases and priming workshops.

Seventy percent of the nurses (35/70) cared for ECMO patients between 2012-2020; 57% took care of more than 10 patients/circuits, 37% between 5 and 10 patients, and only 6% less than 5 patients. More than 88% considered the campus teaching resources, theoretical lessons, and simulation sessions adequate and complete; 94% considered useful in patient care. More than 50% felt well trained in managing ECMO circuit, although some differences according to the scenario were observed (Figure 3). General opinion of the course was: 12% excellent, 51% very good, 34% good, and 2.4% fair. Workshops and clinical cases were the most valued activities of the course (Figure 4).

Since 2006, when the program was launched, 123 patients were assisted with ECMO, for a median time of 3 days (IQR 1-12), median length of stay of 20 days (IQR 1-34) with 54% survival at discharge^[5]^. Twenty-two percent of the patients were neonates, and 11% of the entire population had single-ventricle physiology. Complications were extremely frequent; 72% of the patients had at least one, bleeding in first place (56.9%), and 46% required renal replacement therapy.

We found no significant differences between the two periods. Proportion of neonates was similar in both periods (*P*=0.5), and survival at discharge was slightly higher in 2013-2020 (*P*=0.03), Table 1.

**Table 1:** ECMO survival at discharge, comparison between the two periods.

	2006-2012 (n=35)	%	2013-2020 (n=88)	%	OR (CI 95%)	*P*-value
Neonatal patients	9	25.7	18	20.5		
Pediatric patients	26	74.3	70	79.5	1.3 (0.5-3)	0.5
Survival at discharge	15	42.9	51	58	0.4 (0.18-0.95)	0.03

There were no differences in the proportion of neonates over the two periods. Survival at discharge was higher in 2013-2020.

## DISCUSSION

ECMO is a technically challenging procedure that requires a wide multidisciplinary teamwork to respond with promptness and efficiency, and to deliver continuous care during the whole period of circulatory support. Mechanical complications in ECMO can diminish the survival by up to 71%, therefore, effective training is a necessary strategy to ensure promptness and proper intervention in recognizing and solving problems^[5]^.

Our institution started with an ECMO program supported by certified perfusionists with satisfactory initial results. Facing the increasing number of patients with indication and the severe limitation in the availability of specialist technicians, we developed a simulation-based training program for physicians and nurses with the aim to build an ECMO team to provide proper and qualified response to the new requirements.

Simulation is a well-accepted educational method related to critical care emergencies, team performance, and leadership skills^[6-9]^. It allows students to be centered not only in learning and training technical skills, but also in critical thinking, solving stressful situations, and teamwork in a safe and risk-free environment^[10-13]^. Simulation-based ECMO training has been established as an effective method in initial education and maintenance for both individuals as multidisciplinary teams^[13-16]^. ELSO suggests that institutions developing ECMO programs offer didactic conferences, simulations, and practices with animals to obtain competences.

Considering the evidence about traditional education by learning models that fails when cases are not very frequent or there is a global lack of team experience as in our case, we decided to develop a training program following institutional guidelines^[4]^. The concept of deliberate practice and enhanced performance is supported in multiples domains of experience. Simulation-based training has been shown to improve surgical performance in neck cannulation, in ECMO specialist technical skills and behavior, team performance, attitudes and confidence, and allows an appropriate transfer of those skills to real practice^[17-19]^. This is even more significant in ECMO programs like ours (<20 cases per year) where ELSO recommends complementary continuing medical education to all team members^[4]^. As other published works, simulation scenarios (80%) and priming and troubleshooting workshops were, in our group, the activities considered as essential in maintaining skills^[14-16]^.

The implementation of the simulation-based training program would provide our ECMO team, especially nurses, with the necessary competences to provide continuous care to these critical patients. This training used multiple learning formats, including basic courses with high theoretical content, workshops with traditional simulation and deliberate practice, resolution of high-fidelity health care simulation scenarios with high realism and discussions based on reflection about the practice.

Chan et al.^[19]^ demonstrated in their study about prospective assessment in novices that all participants described didactic education and simulation scenarios as useful to improve their perception on general knowledge, capacity to accomplish critical criteria required in simulated emergencies, and general confidence. Similarly, analyzing our team perception, it was found that more than 80% considered the training highly useful, but we noticed a difference in the perception of it to the different scenarios proposed. This leads us to think about the need to reinforce the features and frequencies of training encounters. A combination of these formats seems to be a proper structure and well-received by the participants. Balancing active repetition with the inherent cognitive load, especially in leadership roles and in those sessions mainly cognitive based, allows for stress reduction and the incorporation of physiological concepts that, finally, must be applied at the patient's bedside. With our program, training not only improved the immediate performance. The follow-up of participants showed the continuous incorporation of the concepts learned and trained during the training to the patient’s real experience. This is proven by survival results and similar complications during the initial implementation in the first years with certified perfusionists.

Lastly, it is important to note that the continuity of the training program is based on ECMO team participants who organize the training of the new ones and oversee periodical training of its members. It is important to mention that outcomes showed no significant differences between the two periods, the first exclusively with perfusionists and the last with specialist nurses caring for the patients. The improvement in survival in the last years is probably due to the increasing experience of the whole team in this procedure. As the main goal of this paper was to present our training program, we did not focus on detailed outcomes that were published in a previous report^[3]^.

## CONCLUSION

In the frame of simulation-based ECMO training program, 24 courses and several continuing teaching activities were carried out over a period of 8 years. Sixty-eight professionals from the PCICU were trained, of which 60% are still part of ECMO team. More than 80% considered the training program as positive, considering workshops and simulation as the most useful components of the program. Professional's confidence in the care of the circuit was greater than in other situations. Since 2013, the program allowed us to provide ECMO to 88 patients with a 58% survival rate at discharge, results within international standards.

## Appendix 1. ECMO training course program.

**Table:**

Theoretical contents	Practical skills	Simulation sessions
ECMO background, physiology, indications, contraindications, Protocol	Equipment recognition	Roles and functions Recognition during connection to ECMO
Roles Circuit monitoring Anticoagulation ECMO patient care	Circuit assembling and priming	Timed assembly and circuit priming
Multiple choice exam	Practical exam	Pump failure Air embolism

Simulation scenarios	Goals
Roles and functions Recognition during admission of an ECMO patient	Leadership during an emergency Recognition of the role of each member Effective communication Teamwork
Equipment assembly and priming	Leadership during an emergency Role recognition: skills, organization, and performance
Pump failure	Problem recognition Effective communication Troubleshooting
Air entry from venous side	Problem recognition Effective communication Troubleshooting
The scenarios were performed in a simulated unit with the same equipment (monitor, ventilator, infusion pumps, defibrillator, emergency medication, etc.). Each scenario was about 15 minutes long, with a final debrief to analyze performance and identify opportunities for improvement.
